# Using natural biostimulants for enhancing defense response of tomatoes against aphids

**DOI:** 10.1371/journal.pone.0340827

**Published:** 2026-01-21

**Authors:** Arij Fakraoui, Zayneb Kthiri, Walid Hamada, Ghazi Krida

**Affiliations:** 1 Laboratory of Bioagressors and Integrated Pest Management in Agriculture (LR14AGR02), National Agronomic Institute of Tunisia (INAT), University of Carthage, Cité Mahrajène, Tunis, Tunisia; 2 University of Carthage, Laboratory of Genetic and Cereal Breeding (LR14AGR01), National Agronomic Institute of Tunisia (INAT), Cité Mahrajène, Tunis, Tunisia; 3 University of Carthage, Higher Institute of Preparatory Studies in Biology and Geology (ISEP BG), Soukra, Tunisia; University of Carthage, TUNISIA

## Abstract

To explore alternative methods for insect control, we investigated the effectiveness of natural biostimulants in triggering defense responses against the cotton-melon aphid *Aphis gossypii* on tomato plants. The tested biostimulants were rosemary essential oil, the fungus *Trichoderma harzianum*, the PGPR (Plant Growth-Promoting Rhizobacteria) *Bacillus subtilis*, and a mix of microalgae. Their effects were compared to an untreated control and to the chemical product Bion (a salicylic acid pathway inducer) as a positive control. Tomato seedlings were exposed to aphid infestation at different leaf levels, and aphid colony development was monitored over time. The impact of biostimulants was assessed by counting aphid numbers at 72 h post-infestation. Additionally, aphid fecundity was evaluated in a subsequent trial with potted tomatoes. In parallel, we explored the rapid effects of root-fed biostimulants on aphid populations using hydroponic tomato seedlings. Leaves from plants treated with biostimulants and with the reference product Bion showed a significant reduction in adult aphid population density and reproduction rates compared to untreated controls. Specifically, control plants exhibited higher aphid reproduction rates, which was significantly reduced at 6 days post-infestation. However, no significant differences were observed between treated and control plants beyond this time, indicating that a strong plant defense response was triggered within one week. Our findings clearly demonstrate the great potential of using biostimulants as promising tools for enhancing tomato integrated pest management.

## Introduction

Tomato (*Solanum lycopersicum* L.), an economically significant and widely consumed solanaceous crop, is a rich source of vitamins, minerals, fiber and antioxidants. It also serves as a plant model for research in functional genomics and plant-pathogen interactions [1,2]. Insect pests, which differ in their capacity to targeting various plant parts (leaves, roots, flowers, fruit, etc.), frequently attack tomato plants on which they induce severe damage. More specifically, aphids (Hemiptera: Aphididae), which are considered among the most economically important sap-sucking insect pests in agriculture, cause significant damage and substantial economic losses to a wide range of crops worldwide [3]. In this context, the cotton-melon aphid *Aphis gossypii* Glover (Hemiptera: Aphididae) has long been considered a widespread and highly destructive pest that poses a significant threat to cultivated plants in Tunisia and worldwide. It causes direct damage by feeding on the plant’s phloem sap and indirectly contributes to greater losses by acting as a vector of viral diseases [4]. Aphids are particularly notorious for their rapid colonization of host plants, a trait facilitated by their reproductive strategy of parthenogenesis, which allows for swift population growth. Their feeding mechanism involves inserting stylets into the plant’s phloem tissue to extract sap [3]. This process not only depletes the plant of essential nutrients but also introduces saliva containing effectors that manipulate plant cellular functions [5,6]. In response, plants have developed various defense mechanisms against these pests [7].

Many studies have explored the mechanisms regulating tomato attractiveness to natural enemies of aphids [8,9]. Flight responses in the aphid parasitoid *Aphidius ervi* Haliday (Hymenoptera: Braconidae), the most effective natural enemy of the potato aphid, *Macrosiphum euphorbiae* (Thomas) (Hemiptera: Aphididae), have been identified through a combination of behavioral assays, tomato volatile organic compound (VOC) analysis, and parasitoid antennal response studies [9]. As part of the plant complex defense response, aphid infestation stimulates the production of methyl salicylate and terpenes [10], suggesting the activation of both the salicylic acid (SA) and octadecanoid (jasmonic acid, JA) pathways. This response shows the potential cross-talk between these signaling pathways, which are primarily associated with plant defense against pathogens and pests, respectively [11]. Plants have evolved a complex, multi-layered defense system in response to aphid infestations. The first line of plant defense involves physical barriers, such as trichomes and cuticular waxes, which impede aphid movement and feeding. Beyond these structural defenses, plants also trigger molecular and biochemical responses [3].

In order to properly manage aphids for enhancing plant protection, it is imperative to investigate novel alternative pest management measures. One promising approach is the use of biostimulants, which act as elicitors to boost plant natural defenses against aphid infestations [6]. These products can be either synthetic (such as fosetyl-Al, Bion, or BABA) or natural (derived from algae, plants, animals, or beneficial microorganisms) [12]. For example, in the last 20 years, plant essential oils (EOs) have attracted considerable attention as potential non-toxic aphicides [13]. Extensive research has also highlighted the role of EOs as plant defense stimulators. For instance, thyme EO has been shown to enhance tomato plant resistance against *Fusarium* disease and gray mold [14]. Moreover, lemongrass EO has been shown to enhance plant defense mechanisms in tomatoes [15]. Meanwhile, numerous studies have focused on the use of seaweed extracts against a range of pests, including sap-feeding hemipterans [16], lepidopterans [17], weevils, termites, and root-knot nematodes. Similarly, brown macroalgae are rich in bioactive chemicals or their precursors, such as alginates [18], laminarins [19], and fucoidans [20]. These bioactive compounds serve as essential elicitors that prime and activate plant defenses [17,21]. For example, in cabbage (*Brassicaeae*), the phenolic compound ekol, isolated from brown seaweed *Ecklonia maxima* (Osbeck) Papenfuss, has been shown to repel the cabbage aphid *Brevicoryne brassicae* (L.) (Hemiptera: Aphididae) [22]. Additionally, some bacterial strains have the ability to trigger plant cellular defense mechanisms against aphids. As an example, root soaking of *Arabidopsis* with *Bacillus velezensis* YC7010 induced systemic resistance against the green peach aphid *Myzus persicae* (Sulzer) (Hemiptera: Aphididae) [23]. Likewise, the treatments of tomato plants with the fungal biocontrol agent *Trichoderma atroviride* strain P1 had a negative impact on the aphid *M. euphorbiae* [24].

In the present study, we aimed to evaluate the potential of several natural biostimulants (rosemary essential oil (EO) (*Rosmarinus officinalis*), the fungus *Trichoderma harzianum*, the PGPR bacterium *Bacillus subtilis*, and a mix of microalgae) as effective organic elicitors capable of activating tomato plant defense mechanisms against *A. gossypii*. This approach is grounded in previous research that has demonstrated the effectiveness of similar biostimulants in enhancing plant defenses against a range of pathogens and aphid species.

## Materials and methods

### Tomato plants

The tomato seedlings, variety “*Savera*” were obtained by sowing seeds in a planting tray filled with peat and maintaining them in a growth chamber (23 ± 2 °C, with a 16h:8h L:D photoperiod) for 30 days. These seedlings served as test subjects to study aphid responses to plants treated with biostimulants after being transferred to plastic pots (6 cm diameter and 11 cm height) filled with a mix of ½ sand, and ½ peat. Throughout their growth, seedlings were watered once a week and once with NPK 20:20:20 fertilizer.

### Aphid infestation and bioassay

*Aphis gossypii* individuals were collected from pepper plants in El Alia (Bizerte, northern Tunisia), and transported in plastic bags to the Laboratory at the National Agronomic Institute of Tunisia where they were maintained under controlled conditions (23 ± 1°C, 60–70% relative humidity, and a 16h:8h L:D photoperiod). On one hand, we maintained the population of *A. gossypii* by transferring to a maximum of five 4–7-day-old female nymphs into a new tomato leaf using a camel hair paintbrush to gently pick them up. On this leaf, the aphid reproduces asexually by releasing live apterous (wingless) nymphs. Later, each rearing leaf was caged in a 9 cm Petri dish. Then, each newborn nymph was transferred into a new leaf, and this procedure was repeated for each new aphid generation. On the other hand, the aphids were massively grown on young seedlings of tomato to preserve the clone throughout the trials.

Aphid populations used in this study originated from a single clonal lineage to minimize genetic variability. This approach ensured a genetically homogeneous population, thereby controlling for interclonal variability in response to biostimulant treatments.

### Assay 1: Application of biostimulants to potted tomato plants

During this study, 5 natural biostimulants were used (Table 1):

**Table 1:** Biostimulants tested in this study, their composition, applied concentrations, and mode of application.

Biostimulant	Composition/ Active ingredient(s)	Applied concentration/ dose	Mode of application	Reference
**Rosemary essential oil (EO)**	Volatile organic compounds (linalool, camphor, geraniol)	10 ppm (10 mg/L solution)	Root feeding (aqueous solution)	[14,15]
***Trichoderma harzianum* (Trianum^®^, Koppert)**	Living spores of *T. harzianum* T22	30 mg/plant	Root feeding (aqueous suspension)	[24]
***Bacillus subtilis* (Serenade^®^, Bayer)**	*B. subtilis* strain QST 713 (≥ 1 × 10⁹ CFU/g)	0.2 mL/plant (suspension)	Root feeding (aqueous solution)	[25]
**Microalgae mix (SynCro™, Algae Energy)**	Mixed microalgae (*Chlorella sp., Scenedesmus sp., Spirulina sp., Synechocystis* sp.)	100 ppm (100 mg/L solution)	Root feeding (aqueous solution)	[12]
**Bion^®^ (Acibenzolar-S-methyl) (Syngenta)**	Synthetic SA analog (50% ASM)	2 mg/plant	Root feeding (aqueous solution)	[8]
**Control**		Distilled water only	Root feeding	

These treatments were used as root feeding; an aqueous solution was prepared by dissolving each treatment in distilled water. The control was treated by the water only. The five biostimulants were applied at the concentrations of 10 ppm/plant for EO, 100 ppm/plant for microalgae, 0.2 ml/plant for the PGPR *B. subtilis*, 30 mg/plant for the fungus *Trichoderma* and 2 mg/plant for Bion.

The applied concentrations and doses were selected based on previous reports and manufacturer recommendations. For rosemary EO, 10 ppm was chosen as it falls within the effective range reported to induce plant defense and reduce insect infestation in tomato and other crops [14,15]. The 100-ppm dose of microalgae was based on earlier studies demonstrating its efficacy in enhancing plant resistance and growth promotion [12]. *For B. subtilis* and *T. harzianum*, the doses correspond to the manufacturers’ instructions (Serenade® and Trianum®, respectively) and are consistent with previous research on induced resistance [11,25]. Finally, the Bion dose (2 mg/plant) was chosen according to Syngenta’s recommendations and based on prior studies on salicylic acid analogs in tomato [8,11].

### Assessment of aphid population density

Four days after the first treatment, 5 one-week-old aphids were placed on the 3^rd^ leaf of each plant. The infested leaves were then covered with plastic and a net trap to avoid any cross-infestation and loss of insects. The number of insect individuals was counted 72 h after the first infestation. The results are presented as the total number of nymphs born from 5 females every 3 days as long as there were surviving larvae or adults. Four days after the second treatment, which occurred 16 days after the first treatment, 5 one-week-old *A. gossypii* individuals were placed on the 5^th^ leaf of each plant. The aphid population density was monitored every three days on treated and control leaves.

### Fecundity assay on tomato plants in pots

The fecundity assay was used to assess the aphid’s ability to reproduce on 30-day-old tomato plants receiving biostimulants. In order to produce a large number of one-day-old nymphs, apterous adult insects (~1–1.5 mm) were brush-applied to tomato leaves one day before starting the experiment. The following day, a single newborn nymph was released on one leaf (3^rd^ stage) of each tomato plant, which was covered with plastic and a net trap to avoid any cross-infestation and loss of insects. Each biostimulant was applied to ten tomatoes, which were considered as replicates. The infested plants were placed inside the growing chamber, where it took approximately 6–8 days for the majority of the nymphs to reach the mature stage and start reproducing. The number of nymphs produced by the mother aphids was determined through a fecundity assay. Afterward, each plant was examined for newly emerged nymphs every two days. Upon counting them, all newly emerged nymphs were removed, leaving only the mother aphids on the plant. The total number of nymphs recovered from each plant during the experiment (11 days) was then calculated. Fecundity was expressed as the average number of nymphs produced daily by each female aphid, which was calculated using the following formula [26]:


Fecundity=N/1(D)

Where, *N* is the total number of newly emerged nymphs recovered from each plant during the experiment and *D* is the total number of days in the experiment. The number *1* indicates the number of insects released on each tomato plant at the beginning of the experiment.

### Assay 2: Application of biostimulants in hydroponic tomato cultivation

Tomato seedlings grown in a hydroponic system were used to assess the effect of biostimulants on the aphid populations. After four weeks of growth in peat, tomato seedlings of the “*Savera”* variety were rinsed at the root level with water to remove the peat. Eight seedlings were then transferred into plastic box filled with one liter of Hoagland nutrient solution (3.03 g.L^-1^of KNo_3_, 1.15 g.L^-1^ of NH_4_H_2_PO_4_, 1.23 g.L^-1^ of MgSO_4_, 7H_2_O, 1.30 g.L^-1^ of Ca(NO_3_)_2_, 4H_2_O, 168 mg.L^-1^ of KCL, 77.3 mg.L^-1^ of H_3_BO_3_, 22 mg.L^-1^ of MnSO_4_, 4H_2_O, 29 mg.L^-1^ of ZnSO_4_, 7H_2_O, 6 mg.L^-1^ of CuSO_4_, 5H_2_O, 36 mg.L^-1^ of H_2_MoO_4_, 4H_2_O and 5 ml.L^-1^ of Fe EDTA). The seedlings within the plastic boxes were kept in a growth chamber with 16 h of light and 8 h of darkness at 22°C. Air pumps were used to aerate the seedlings roots. The following biostimulants were applied into the Hoagland solution and supplied to the tomato plants through root feeding: rosemary EO (10 µl/L), microalgae (100 µl/L), *B. subtilis* (2 ml/L), *T. harzianum* (300 mg/L), and Bion (8 mg/L) in addition to an untreated control. For each treatment, three replicates were considered, consisting of three boxes with 8 seedlings. One aphid individual was placed on each plant within the box and covered with plastic and a net trap to avoid any cross-infestation and loss of insects. The growth and fecundity rates of aphids were assessed at 24-, 48-, 72-, and 120-hours after their introduction on host plants.

### Statistical analyses

Statistical analyses were performed using the software SPSS version 27. Data was subjected to a normality test. The variance was analyzed using one-way ANOVA. The model used was as follows:


$${\text{Y}}_{\text{ijkl}}=\mu +{\text{T}}_{\text{i}}+{\text{D}}_{\text{j}}+\text{D}*{\text{T}}_{\text{k}}+{\text{e}}_{\text{ijkl}}$$

Where: Y_ijkl_ = variable of interest; μ = population mean, T_i_ = fixed effect of treatment (i = 1, 6), D_j_ = fixed effect of date (j = 1–4), e_ijkl_ = residual error (0, σ_2_e).

### Ethics statement

This study was conducted in compliance with all applicable ethical guidelines for research involving plants and insects. The experimental protocols were reviewed and approved by the National Institute of Agronomy of Tunisia. Since this research involved aphid infestations on tomato plants, no specific human or animal ethics approval was required. The aphids (*Aphis gossypii*) were collected under standard ecological research practices, ensuring no harm to native populations. No informed consent was applicable for this study, as it did not involve human participants or vertebrate animals.

## Results

### Effect of the biostimulants on aphids on tomato plants maintained in pots

**Fig 1:**
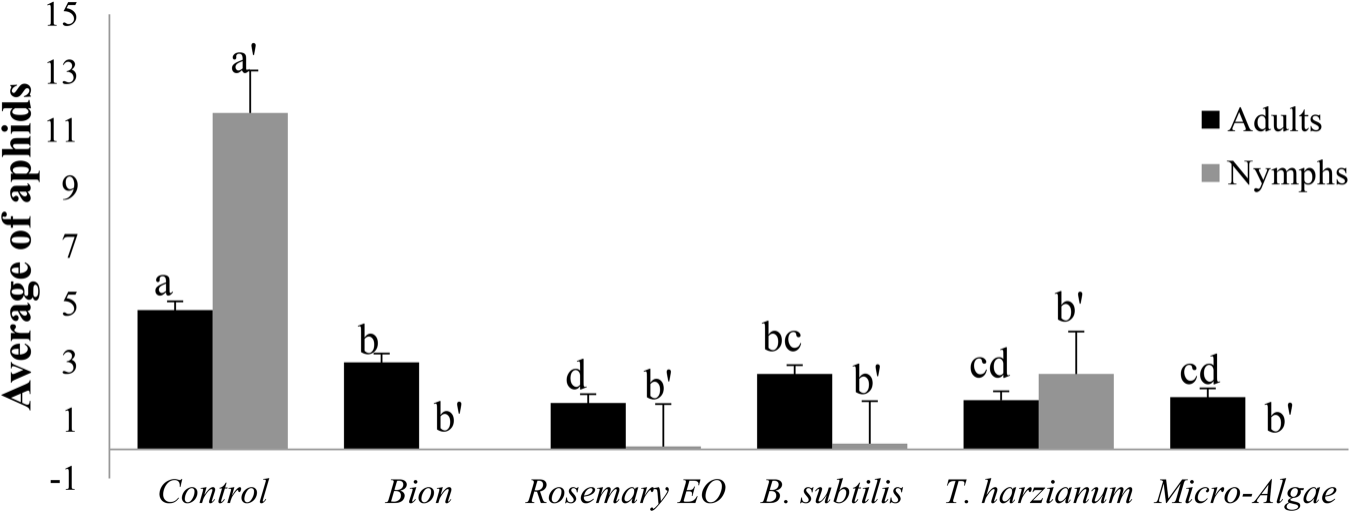
Effect of the treatment on the average number of aphids adults and nymphs. Four days after treatment, each pot was infested with 5 adult aphids that are 7-day-old at the third leaf level. The aphid assessment was made 3 days after infestation.

The first treatment assessment, conducted 3 days after the initial infestation, revealed a clear distinction between control and treated plants. All treatments significantly reduced aphid populations density compared to the control (Fig 1, Table 2). Bion achieved a reduction of approximately 83% in nymphs and 67% in adults. Rosemary essential oil (EO) demonstrated the highest efficacy, with a near-total elimination of nymphs (nearly 100%) and an 83% reduction in adult aphid numbers. *Bacillus subtilis* and microalgae resulted in moderate reductions, decreasing nymph and adult populations by 92% and 67%, respectively. *Trichoderma harzianum* showed slightly lower efficacy, reducing nymphs by about 75% and adults by 50%.

**Table 2:** One-way ANOVA of the effect of the biostimulants on aphid adults and nymphs (C + 3, T + 7).

ANOVA	ddl	Adults	Nymphs
Treatments	5	74,483***	871,333***
Error	54	50,100	1155,400

The sum square values with statistical significance are shown (ns: non-significant, *: p < 0.05; **: p < 0.01; ***: p < 0.001).

The control group displayed the highest average number of aphid populations, with approximately 6 adults and 13 nymphs, while all treatments, including Bion, Rosemary EO, *B. subtilis*, *T. harzianum*, and microalgae, reduced aphid populations (nymphs and adults) to nearly zero. This result highlights the effectiveness of all treatments in significantly reducing aphid populations (Table 3).

**Table 3:** One-way ANOVA of the effect of the biostimulants on aphid adults and nymphs (C + 6, T + 10).

ANOVA	ddl	Adults	Nymphs
Treatments	5	5.333***	249.950***
Error	54	5.600	264.900

The sum square values with statistical significance are shown (ns: non-significant, *: p < 0.05; **: p < 0.01; ***: p < 0.001).

**Fig 2:**
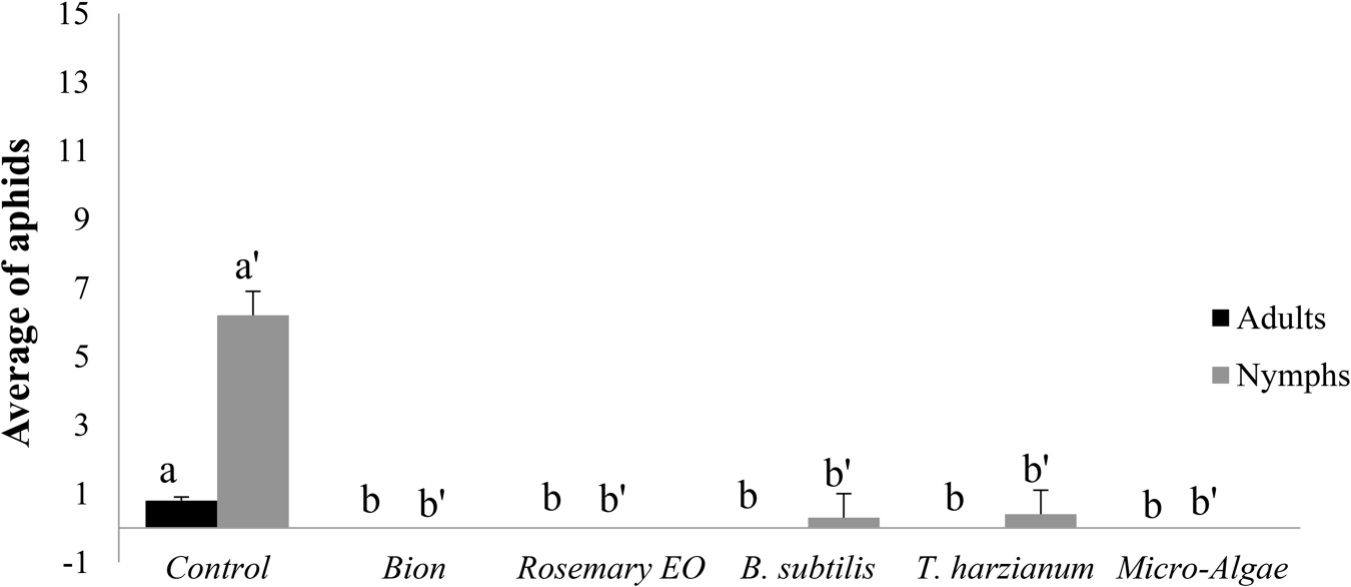
Effect of treatment on the average number of aphid adults and nymphs. Four days after treatment, each pot was infested with five 7-day-old adult aphids at the third leaf level. The aphid density assessment was made 6 days after infestation.

The number of aphids decreased in all treatments, including the untreated control (Figs 1 and 2). However, all treatments (Bion, Rosemary EO, *B. subtilis*, *T. harzianum*, and microalgae) significantly reduced aphid populations (nymphs and adults). This underscores the effectiveness of these treatments in reducing aphid infestations compared to the untreated control.

**Fig 3:**
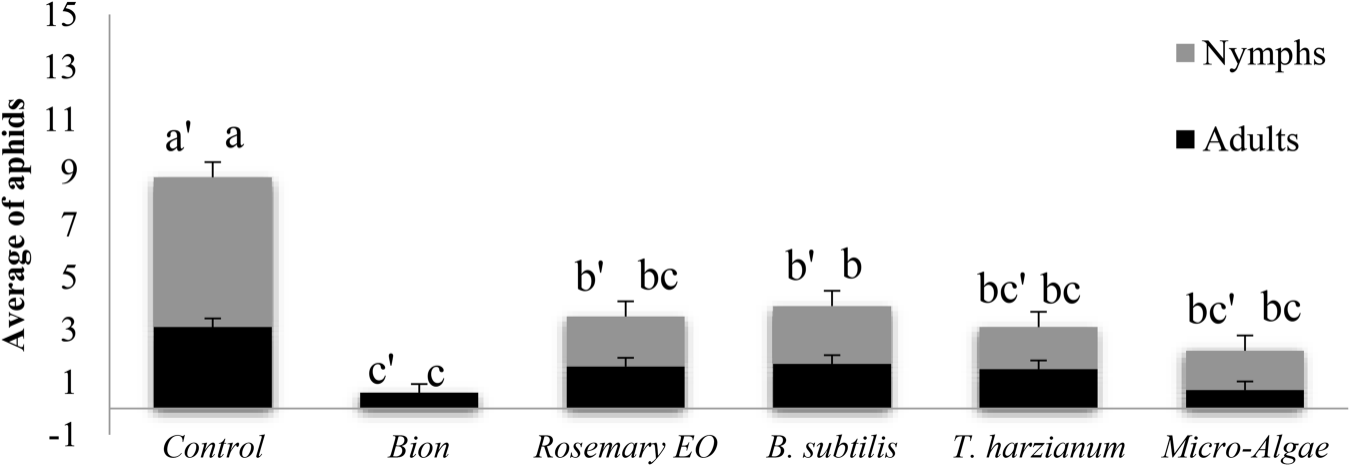
Effect of the second treatment on the average number of aphid adults and nymphs. Four days after the second treatment, each pot was infested with five 7-day-old adult aphids at the third leaf level. The aphid density assessment was made 3 days after infestation.

Regarding the second treatment assessment, our results demonstrated a significant reduction in aphid population numbers following the application of biostimulant treatments compared to the untreated control (Table 4). In the control group, the average number of newborn nymphs and adults was 5.7 and 3.1, respectively. Treatments significantly decreased these numbers, with Bion showing the highest effectiveness by completely eliminating nymphs and reducing adults to 80.6%. Microalgae also exhibited high effectiveness, reducing nymphs by 73.7% and adults by 77.4%. Similarly, *T. harzianum* and rosemary EO decreased nymphs by 71.9% and 66.7% and adults by 51.6% and 48.4%, respectively. *B. subtilis* was the least effective, reducing nymphs by 61.4% and adults by 45.2%. These findings highlight the effectiveness of biostimulants, particularly Bion, as sustainable tools for controlling aphid populations, offering substantial reductions in aphid numbers compared to untreated plants (Fig 3).

**Table 4:** One-way ANOVA of the effect of the biostimulants on aphid adults and nymphs (C_2_ + 3, T_2_ + 7).

ANOVA	ddl	Adults	Nymphs
Treatments	5	40.533***	180.150***
Error	54	58.400	181.500

The sum square values with statistical significance are shown (ns: non-significant, *: p < 0.05; **: p < 0.01; ***: p < 0.001).

**Fig 4:**
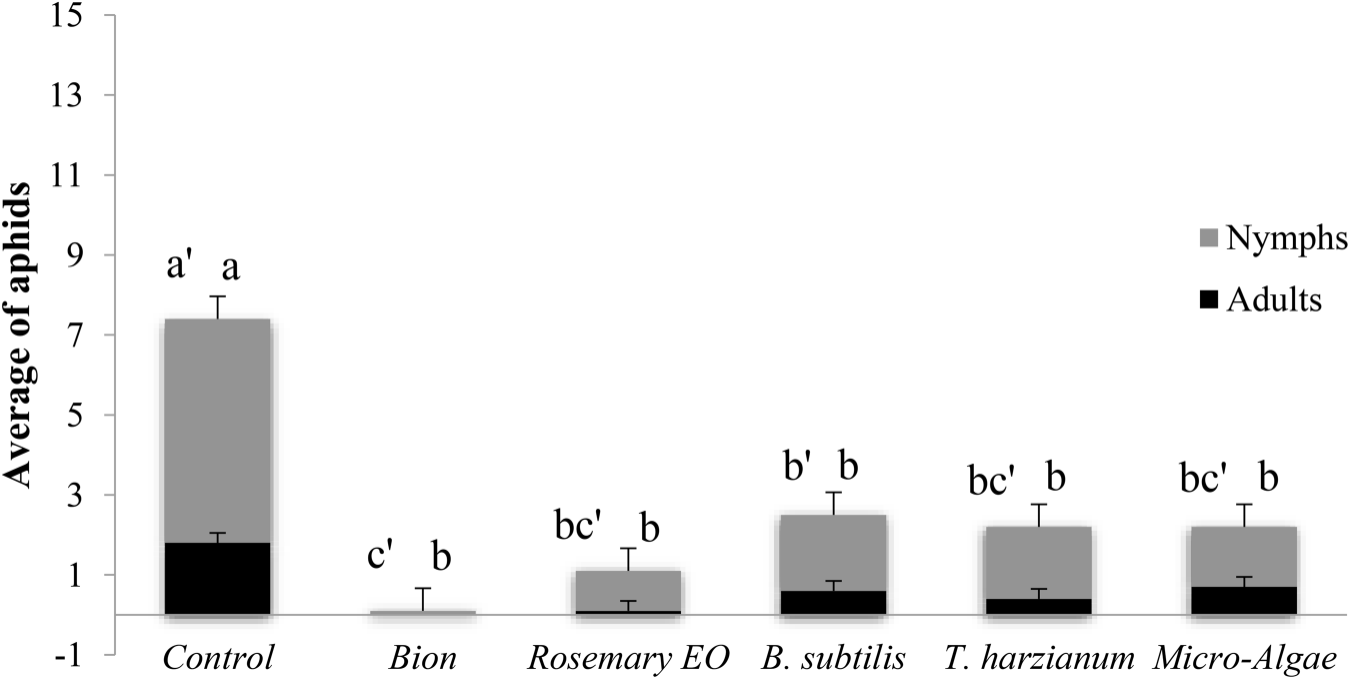
Effect of the second treatment on the average number of aphid adults and nymphs. Four days after the second treatment, each pot was infested with five 7-day-old adult aphids at the third leaf level. The aphid density assessment was made 6 days after infestation.

Aphid numbers were lower in the second treatment assessment (Fig 4). There was a highly significant difference (P < 0.001) in the number of adults and nymphs between the plants treated with biostimulants and the untreated plants (Table 5). The second treatment revealed that the biostimulants’ efficacy was only temporary, as it lasted no more than ten days.

**Table 5:** One-way ANOVA of the effect of the biostimulants on aphid adults and nymphs (C_2_ + 6, T_2_ + 10).

ANOVA	ddl	Adults	Nymphs
Treatments	5	21.000***	178.683***
Error	54	33.400	172.300

The sum square values with statistical significance are shown (ns: non-significant, *: p < 0.05; **: p < 0.01; ***: p < 0.001).

As shown in Fig 4, this result illustrates the average number of aphids (adults and nymphs) across different treatments. The control group showed the highest aphid population density, with an average of 7 nymphs and 3 adults. The Bion treatment was the most effective, with almost no nymphs, corresponding to a reduction of over 90% compared to the control. Rosemary EO, *B. subtilis*, *T. harzianum*, and microalgae treatments showed significant reductions in aphid populations density, reducing the total aphid count to intermediate levels of approximately 2–3 on average, with no statistical differences among them. These results highlight the potential of biostimulants, especially Bion, for effectively controlling aphid populations by significantly reducing both nymph and adult counts compared to untreated plants.

### Effect of the biostimulants on aphid fecundity on tomato plants maintained in pots

**Fig 5:**
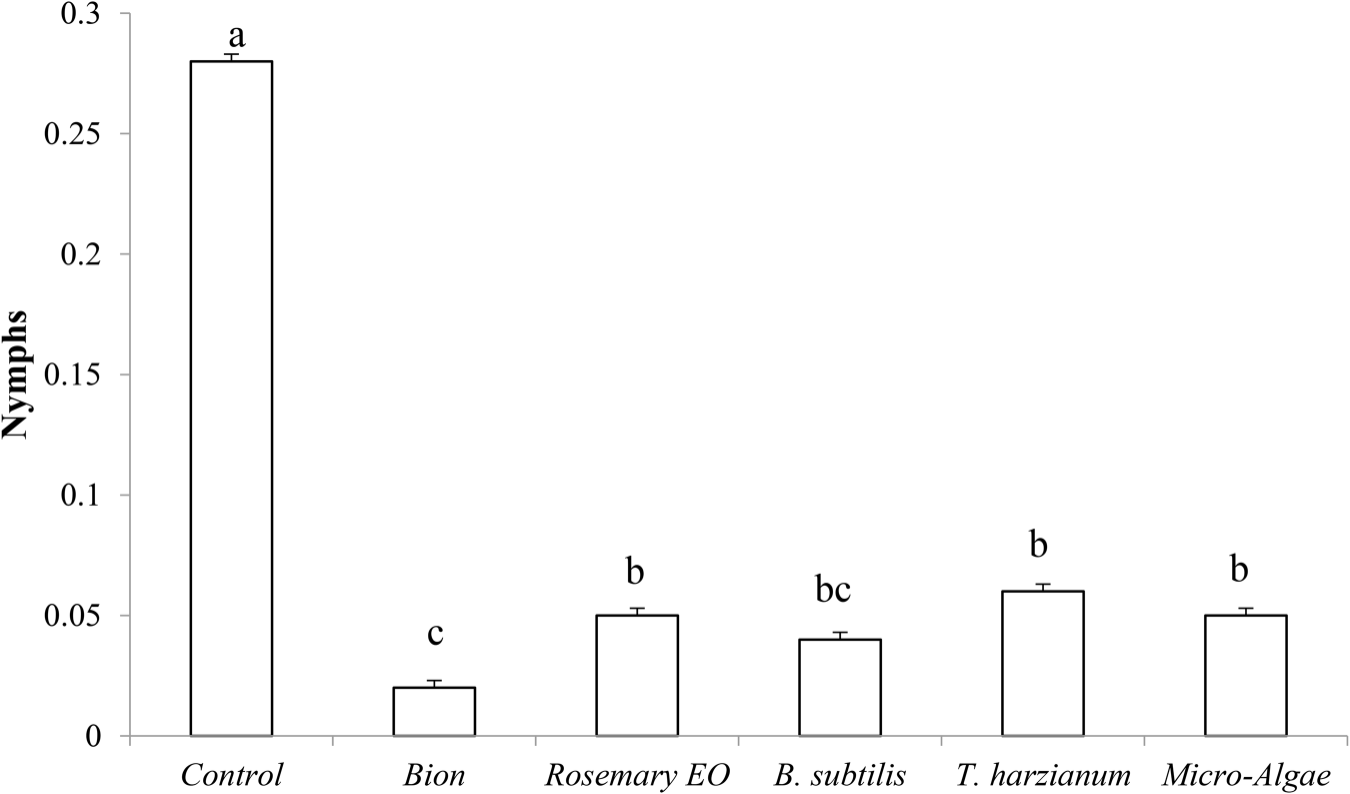
Treatment impact on the average number of aphid nymphs/mother aphids over a period of 11 days on untreated and treated plants (n = 10). Values with different letter are significantly different at p < 0.005.

According to the ANOVA results (Fig 5), the difference in fecundity between untreated and biostimulant-treated plants was significant (P < 0.05).

The results revealed significant differences in number of nymphs across treatments. The control group showed the highest nymph count (approximately 0.3), which was significantly higher than all treatments. The Bion treatment was the most effective, reducing the nymph population by 83% compared to the control. Rosemary EO, *T. harzianum*, and microalgae treatments reduced the nymph count to similar intermediate levels (0.1), showing approximately 66% reduction compared to the control. *B. subtilis* also reduced nymph numbers but to a slightly lesser extent. These findings highlight the effectiveness of biostimulants, particularly Bion, in significantly decreasing the nymph population.

### Effect of the biostimulants on aphids on tomato plants maintained in hydroponic system

The hydroponics experiment was conducted because it facilitates faster and more effective absorption of biostimulants. Aphid growth and productivity were examined during a time course of 24, 48, 72, and 120 h after infestation, as shown in Tables 6 and 7 below.

**Table 6:** Number of aphids (adults and nymphs) (mean ± SE) on each tomato plant maintained in hydroponics and root-fed by biostimulants compared to untreated plants.

Treatment	24h	48h	72h	120h
**Untreated control**				
Adults	3 ± 0 a	3 ± 0 a	3 ± 0 a	2 ± 0 a
Nymphs	1 ± 1.73 a’	2 ± 2.65 a’	2 ± 2.65 a’	7 ± 1 a’
**Bion**				
Adults	1.67 ± 0.58 b	1.33 ± 0.58 c	1.33 ± 0.58 c	1 ± 1a b
Nymphs	0.67 ± 1.15 a’	0.67 ± 1.15 a’	0.33 ± 0.58 a’	0 ± 0 b’
**Rosemary EO**				
Adults	3 ± 0 a	2.67 ± 0.58 ab	2 ± 0 b	0.67 ± 0.58 b
Nymphs	0 ± 0 a’	0 ± 0 a’	1 ± 1 a’	1.33 ± 1.53 b’
***B. subtilis***				
Adults	2 ± 0 ab	2.67 ± 0.58 ab	1.67 ± 0.58 bc	1.33 ± 0.58 ab
Nymphs	0 ± 0 a’	0.67 ± 1.15 a’	1.33 ± 2.30 a’	1.33 ± 1.16 b’
***T. harzianum***				
Adults	2.33 ± 0.58 ab	2 ± 0 bc	2 ± 0 b	1 ± 0 ab
Nymphs	0 ± 0 a’	0 ± 0 a’	0.67 ± 1.15 a’	1 ± 1.73 b’
**Microalgae**				
Adults	2.33 ± 1.15 ab	2.33 ± 0.58 ab	2 ± 0 b	1.67 ± 0.58 ab
Nymphs	0 ± 0 a’	0 ± 0 a’	0 ± 0 a’	0 ± 0 b’

For adult aphids, means followed by the same small letter are not statistically different in each column and values followed by the same small letter with a coma are not significantly different in each column for aphid nymphs (Tukey’s HSD, P < 0.05).

**Table 7:** One-way ANOVA of the effect of the biostimulants on adults and nymphs of aphids on tomato plants maintained in hydroponics.

	ANOVA	ddl	Adults	Nymphs
25h	Treatments	5	4.278ns	2.944ns
	Error	12	4.000	8.667
48h	Treatments	5	5.333*	9.111
	Error	12	2.667	19.333
72h	Treatments	5	4.667**	7.778
	Error	12	1.333	30.000
120h	Treatments	5	3.611	103.778***
	Error	12	4.000	15.333

The sum square values with statistical significance are shown (ns: non-significant, *: p < 0.05; **: p < 0.01; ***: p < 0.001).

After 120 h, the biostimulants demonstrated a significant effect on the nymph population (p < 0.001). However, for adult aphids, a significant difference was observed only after 72 h. However, after 24 h, the three adults placed on the untreated plants survived and even reproduced, with an average of 1 nymph (Table 6). In the untreated control, the population density averaged 3 adults and reached 7 nymphs after 120 h. In contrast, Bion showed the highest efficacy, significantly reducing both adults and nymphs over time, with no nymphs and only 1 adult observed at 120 h. Rosemary EO was also effective, keeping low adult numbers (0.67–2) and reducing nymphs to 1.33 in 120 hours. *B. subtilis* and microalgae treatments showed intermediate efficacy, with adult counts ranging between 1.67 and 2.33 and nymphs reduced to 1.33 at later time points. *T. harzianum* exhibited a moderate reduction, particularly in nymphs, which remained below 1. These results demonstrate the effectiveness of biostimulants in reducing aphid populations over time, with Bion and rosemary EO showing the most important reductions.

## Discussion

The induced defense response by biostimulants in plants is well documented for its ability to trigger defense mechanisms against several pathogens, including fungi and bacteria [8,11]. However, little is known about their capacities to induce protective reactions against insects. Therefore, this study investigated the potential of different biostimulants: rosemary EO, the fungus *T. harzianum*, the PGPR *B. subtilis*, a mix of microalgae, and the reference product as natural alternatives to synthetic pesticides.

All biostimulants significantly reduced aphid populations compared to the untreated control, though their efficacy varied among products [27]. Both Bion and the natural biostimulants were effective, particularly within the first 7–10 days after treatment. However, aphid numbers gradually declined in all treatments, including the control, and biostimulant efficacy diminished after 10 days, suggesting the need for reapplication. Overall, the tested biostimulants played an important role in limiting aphid proliferation. The higher efficacy of rosemary EO and microalgae in reducing aphid populations could be attributed to their unique mechanisms of action. EOs, like rosemary oil, contain volatile organic compounds (VOCs), such as linalool, camphor, and geraniol, which act as insect repellents or disrupt aphid behavior [16,28]. Additionally, these VOCs may function as semiochemicals, interfering with aphid-host recognition [14]. Beyond their direct effects on aphids, EOs play a crucial role in modulating plant defense mechanisms [29]. They have been shown to activate key signaling pathways associated with salicylic acid (SA) and jasmonic acid (JA), two major plant hormones involved in stress responses [30]. The activation of these pathways enhances systemic acquired resistance (SAR), priming the plant to respond more effectively to pest attacks [14]. This dual mode of action direct toxicity or repellency to aphids and indirect enhancement of plant defenses suggests that rosemary EO could serve as an effective, eco-friendly alternative to synthetic insecticides.

Similarly, microalgae-based biostimulants contribute to aphid control through both direct and indirect mechanisms. Microalgae contain bioactive compounds such as polysaccharides, fatty acids, and antioxidants, which can improve plant health, enhance resistance to pests, and even produce allelopathic effects that deter aphid settlement [31]. In addition, microalgae stimulate plant growth and vigor by improving nutrient uptake and enhancing stress tolerance, further reinforcing the plant’s natural defenses against aphid infestation [32]. On the other hand, the microalgae mixture might exert its effects by enhancing the nutrient content and systemic defenses in plants, as suggested by its ability to upregulate genes like PR-1 and PAD3, which are associated with plant defense pathways [28]. These metabolites may improve the plant’s ability to produce toxic compounds, further reducing aphid colonization. Further studies could investigate how these specific metabolites contribute to the observed differences in biostimulant efficacy [28].

Moreover, the findings from hydroponic trials corroborated the results from pot experiments, confirming that aphid populations were consistently lower in plants treated with biostimulants compared to untreated controls and those treated with the reference product (Bion). This suggests that biostimulants trigger systemic defenses against aphids in tomatoes by inducing the release of elicitor chemicals. These elicitors stimulate the production of metabolites in plants, which may have repellent, or toxic properties against insects. In the first trial, tomato plants treated with rosemary EO, both in pots and hydroponics, showed a greater reduction in aphid populations than those treated with other biostimulants or the reference product. Research on EOs has demonstrated their effectiveness in inducing plant resistance. For instance, *Cymbopogon citratus* EO significantly reduced cases of fusarium wilt and showed resistance-inducing properties against fungal pathogens like *Botrytis cinerea* [14]. Similarly, Abu Alfayah [28] reported that rosemary EO exhibited insecticidal activity against *Myzus persicae* (Sulzer) and could naturally induce resistance in potatoes, which was also the case in our study.

The second product in efficacy was the microalgae mix, which significantly reduced aphid numbers in the first trial compared to untreated plants and other biostimulants. Notably, aphid nymph populations totally disappeared within seven days of treatment in hydroponic systems, as observed with the reference product (Bion). The role of bioavailability and persistence of microalgae formulations in hydroponic and soil-based systems warrants further research. These formulations might offer prolonged efficacy compared to EOs due to their nutrient-enrichment properties, which could enhance systemic defense over time. Although research on PDS (plant defense stimulator) compounds in microalgae is in its early stages, studies suggest that microalgae treatments can upregulate defense-related genes, such as PR-1, PAD3, ACS6, and WRKY 40 [33,34].

The third most effective treatment was either *T. harzianum* or *B. subtilis,* which significantly reduced aphid populations compared to untreated controls. These biostimulants demonstrated efficacy in both pot and hydroponic systems, similar to the reference product. In fact, the genus *Trichoderma* includes PGPFs (Plant Growth-Promoting Fungi), renowned for their biocontrol properties and ability to enhance plant resistance against pathogens. *T. harzianum* can trigger ISR (Induced Systemic Resistance) in plants, activating defense pathways that enhance resistance to a broad spectrum of pathogens. This systemic resistance may also influence herbivorous insects like aphids by making the plant less palatable or suitable for feeding [35]. Also, *T. harzianum* produces secondary metabolites with antimicrobial properties, inhibiting the growth of soil-borne pathogens. By maintaining plant health, these antifungal compounds indirectly reduce the susceptibility of plants to secondary infestations, including those by aphids [35]. Similarly, *Bacillus* species have been shown to enhance plant defense by regulating reactive oxygen species (ROS) production, upregulating defense-related genes, and stimulating phytohormone pathways such as salicylate and jasmonate [36]. On the other hand, *B. subtilis* produces lipopeptides with antimicrobial properties, which can suppress pathogenic microbes. A healthier plant with a robust microbial community is less attractive to aphids, potentially reducing their infestation rates [25].

The observed effects of biostimulants on both pot-grown and hydroponic tomato plants underscore their potential to effectively reduce aphid populations densities, likely through systemic activation of plant defenses. The ability of hydroponic system to enhance biostimulant uptake further validated the efficacy observed in pot trials. The tripartite interaction between biostimulants, tomatoes, and aphids induces overexpression of genes involved in hormonal signaling pathways, including salicylic acid (SA), jasmonic acid (JA), and ethylene (ET), which plays a key role in activating systemic acquired resistance (SAR) and priming the plants for enhanced defense responses. The presumed mechanisms of action of the tested biostimulants differ but collectively enhance tomato resistance to aphids. Rosemary EO acts both directly, through volatile compounds such as linalool and camphor that repel or disrupt aphid behavior [16,28], and indirectly by activating salicylic acid (SA) and jasmonic acid (JA) signaling pathways involved in systemic acquired resistance [14,29,30]. The microalgae mix provides bioactive molecules (e.g., polysaccharides, fatty acids, antioxidants) that stimulate metabolism, improve nutrient status, and upregulate defense-related genes such as PR-1 and PAD3 [31,34]. *T. harzianum* enhances induced systemic resistance (ISR), activating defense signaling and producing secondary metabolites that make plants less suitable for herbivores [35]. Similarly, *B. subtilis* promotes induced resistance by regulating reactive oxygen species (ROS), modulating SA and JA pathways, and producing antimicrobial lipopeptides that indirectly limit pest colonization [25,36]. Finally, the reference product Bion (acibenzolar-S-methyl) mimics SA activity, activating systemic acquired resistance (SAR) and priming plants against subsequent attacks [10,11]. Together, these distinct but complementary mechanisms highlight the potential of biostimulants as sustainable alternatives for integrated pest management.

Biostimulants trigger molecular alarm cascades, enhancing the plant’s ability to activate rapid and effective defenses against subsequent pest challenges. This boosts the plant’s resilience to external threats from pests [10]. Furthermore, their incorporation into integrated pest management (IPM) systems provides a sustainable alternative by reducing dependence on chemical pesticides, thereby minimizing the risk of resistance development in aphid populations.

## Conclusion

This study demonstrated that natural biostimulants applied to tomato roots effectively reduced populations of *A. gossypii* adults and nymphs, likely through induced defense mechanisms similar to those triggered by the salicylic acid (SA) pathway, as observed with the reference product. Further analyses on hormonal signaling (SA, JA, ABA, and ethylene), gene expression, and defense-related metabolites (e.g., terpenoids, (E)-β-farnesene) are needed to clarify the mechanisms underlying aphid resistance, including studies on hormone-deficient tomato mutants. From a practical standpoint, rosemary EO, microalgae formulations, *T. harzianum*, and *B. subtilis* could be integrated into pest management programs as eco-friendly alternatives to synthetic insecticides. Their use may reduce chemical inputs, delay aphid resistance development, and support sustainable tomato production, although repeated applications may be required to maintain their effectiveness. These findings highlight the great potential of biostimulants as promising, sustainable tools for enhancing defense response of tomatoes against aphids.

## Supporting information

S1 File Supporting information. (XLSX)
